# Word Length in Political Public Speaking: Distribution and Time Evolution

**DOI:** 10.3390/e26030180

**Published:** 2024-02-21

**Authors:** Natalia L. Tsizhmovska, Leonid M. Martyushev

**Affiliations:** 1 Technical Physics Department, Ural Federal University, 19 Mira St., 620002 Ekaterinburg, Russia; n.l.tsizhmovska@urfu.ru; 2 Institute of Industrial Ecology, Russian Academy of Sciences, 20 S. Kovalevskaya St., 620219 Ekaterinburg, Russia

**Keywords:** quantitative linguistics, word lengths, principle of least effort, lognormal distribution, brevity and Menzerath–Altmann laws

## Abstract

In this paper, word length in the texts of public speeches by USA and UK politicians is analyzed. More than 300 speeches delivered over the past two hundred years were studied. It is found that the lognormal distribution better describes the distribution of word length than do the Weibull and Poisson distributions, for example. It is shown that the length of words does not change significantly over time (the average value either does not change or slightly decreases, and the mode slightly increases). These results are fundamentally different from those obtained previously for sentence lengths and indicate that, in terms of quantitative linguistic analysis, the word length in politicians’ speech has not evolved over the last 200 years and does not obey the principle of least effort proposed by G. Zipf.

## 1. Introduction

Natural languages have been one of the most important subjects of science for a long time. However, the interest in language research has flared up with renewed vigor in recent times due to the development of computer technology, general digitalization, development of artificial intelligence systems, etc. One of the scientific areas demonstrating a new impetus for development is quantitative linguistics. Here, quantitative statistical methods of analysis are used to study texts and speeches, which helps to gain a deeper understanding of the structure and evolution of language. By transforming linguistic objects of natural language into numbers according to certain rules, quantitative linguistics strives, on the basis of statistical analysis, to formulate general laws according to which language functions. There are a number of achievements along this path, three of which are as follows: Zipf’s law: the frequency of words is inversely proportional to their rank in frequency lists. Brevity law: the more frequently a word is used, the “shorter” that word tends to be. Menzerath–Altmann law: the sizes of the constituents of a construction decrease with the increase in size of the construction under study, and vice versa [1,2].

It is well known [3,4] that homogeneous and representative datasets (collections of texts) should be used to detect linguistic laws, since even the genre of the text [5,6,7], not to mention the time of its writing, can influence the results obtained. An important dataset for study is the texts of politicians’ public speeches. These texts are well documented over an interval of sufficient length and “ought to be fairly consistent in genre and register” [8]. An important feature of political speeches is that for the most part they are carefully prepared for perception and effective impact on the average mass listener living in a certain time epoch. Therefore, despite the specific genre, political speeches are an excellent object for studying the laws and evolution of language.

The study of politicians’ public speeches using the methods of quantitative linguistics has been previously carried out in works [8,9,10,11,12]. In particular, [8] analyzes the inaugural speeches of USA presidents and notes that the average length of sentences has monotonically decreased over 200 years, and in total has decreased by approximately 50%. In [9], this conclusion for the USA was confirmed and also generalized to public speeches by members of UK parliamentary parties in the period from 1900 to 2000. It was found in [9] that the frequency distribution of sentence lengths during public speaking in the USA and the UK is best described not by the lognormal distribution, but by the Weibull distribution. A similar distribution and reduction in the length of sentences over time was associated in [9] with the principle of least effort proposed by G. Zipf [4,13,14]. The formulation of this principle is as follows: “… the person will strive to minimize the probable average rate of his work-expenditure (over time). And in so doing he will be minimizing his effort. … Least effort, therefore, is a variant of least work.”.

Will the length of words, rather than sentences, also obey the principle of least effort for the same public speeches of politicians, i.e., have Weibull distributions and decrease over time?

This is a very interesting and controversial issue. Indeed, according to Zipf’s law and the brevity law, the power function describes the distribution of word lengths [13,14,15] and considering the Weibull distribution is an unnecessary complication. However, a question arises regarding the validity of these laws when considering words of arbitrary length. For example, according to studies [15] conducted for English and Swedish, only words containing more than three letters follow this law and without a lower bound on word length, the distribution of word lengths should be more complex. Both discrete distributions (especially Poisson) [4,5] and continuous distributions (in particular, lognormal and gamma distributions) [13,15,16,17,18] have been previously proposed in the literature as possible options. Regarding the temporal behavior of average word length, research results are very contradictory [5]. Thus, according to the Menzerath–Altmann law, as the length of sentences calculated in words decreases, the length of the words should increase on average. From the studies of politicians’ public speeches [8,9,10], the sentence length, as mentioned above, decreases significantly over time. However, according to [8], the length of the word does not increase, but rather decreases slightly (by about 5% over the last century). Note that for other genres of text, there has been a slight increase in the average word length over several centuries for Arabic [7], as well as for English and Russian throughout the nineteenth and most of the twentieth century [11] (however, according to [11], since the end of the twentieth century, there has been a decrease in the average word length). Other studies of changes in average word length over time can be found in [5,11,19]; their results are extremely contradictory. Thus, as seen here, there is no answer to the question formulated above. The purpose of this paper is to attempt to answer it within quantitative linguistics.

## 2. Data for Analysis

The choice of public speeches of politicians in the USA and the UK was determined by the availability of speeches in digital archives, as well as by a desire for homogeneity of texts by genre and uniformity in distribution over time.

We analyzed text transcripts of 88 speeches by USA presidents from 1789 to 2021 (including 59 inaugural addresses), available in [20,21]. Six speeches were made in the 18th century, 51 speeches were made in the 19th century, 25 in the 20th century, and 6 in the 21st century. For the 18th and 19th centuries, speeches by USA presidents were both inaugural and other speeches. These speeches were made in 1789, 1793, 1796–1798, 1801, 1803, 1805, 1809, 1812, 1813, 1815, 1817, 1821, 1825, 1827, 1829, 1833, 1837–1839, 1841, 1842, 1845, 1846, 1848–1851, 1853, 1854, 1856, 1857, 1861, 1864, 1865, 1869, 1873, 1875–1877, 1879, 1881, 1885, 1889, 1893, 1895, 1897. For the 20th and 21st centuries, only inaugural addresses were used (except for 1968, where an additional speech by President Johnson was used); as a result, they are uniformly distributed every four years 1901–2021.

For the UK, transcripts of 247 speeches were analyzed for the period from 1808 to 2018. These speeches are available in [22,23]. Initially, only the speeches of UK party leaders were considered, but they only covered the period from 1895 to 2018. To extend the temporal coverage, speeches by queens and kings have been added, as well as some additional speeches by party members in Parliament. As a result, 19 speeches were analyzed for the 19th century (they belonged to 1808, 1814, 1814, 1815, 1817, 1819, 1827, 1830, 1837, 1842, 1853, 1868, 1877, 1893, 1895, 1896, 1897, 1897, 1899), 170 for the 20th century (1900–1913, 1917–1930, 1932–1937, 1941–1943, 1945–1951, 1955–1958, 1960–1999), and 58 speeches (2000–2018) for the 21st century.

The word length was determined by the number of letters from space to space. This approach is not universal, but it is widely used [19,24]. It is known that there is still no universal, generally accepted unit for measuring word length (number of syllables, number of letters, breath groups, etc.). The choice of such a unit is made on the basis of the problem statement [4,19,24]. At the moment, we cannot be completely sure that the method of measuring word length does not affect the results obtained. So, in [25], it is stated that: “There can be no a priori decision as to what a word is, or in what units word length can be measured. Meanwhile, in contemporary theories of science, linguistics is no exception to the rule: there is hardly any science which would not acknowledge, to one degree or another, that it has to define its object, first, and that constructive processes are at work in doing so. What has not yet been studied is whether there are particular dependencies between the results obtained on the basis of different measurement units; it goes without saying that, if they exist, they are highly likely to be language-specific.”.

**Figure 1:**
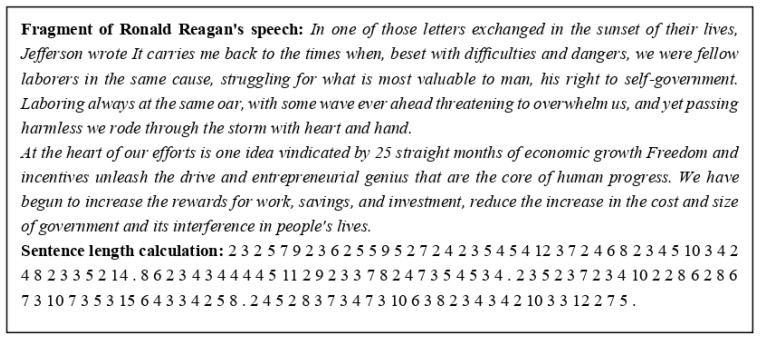
Example of the word length calculation by the algorithm used in the study.

Word length calculation was performed automatically using the developed and tested computer program (see an example of such calculation in Figure 1).

As can be seen from Figure 1, the software we use counts digits, and this can often be incorrect in terms of the length of the spoken words that these digits represent. We did not change the software algorithm since this is a rather non-trivial computer task. But the most important reason for our inaction in this matter is that the error introduced by these digits into the results obtained in the paper turns out to be negligibly. This is due to the fact that the share of the number of digits in relation to the total number of words in the studied political speeches is very small. In the speech, a fragment of which is presented in Figure 1, this share is 0.003, and the highest share in the studied speeches was about 0.004. The statistical quantities studied below are not sensitive to this influence. This software was previously used to calculate sentence lengths as well [9]. Therefore, the dots in the text are saved after processing, as is demonstrated in the fragment below. These dots have no effect on word length processing.

**Figure 2:**
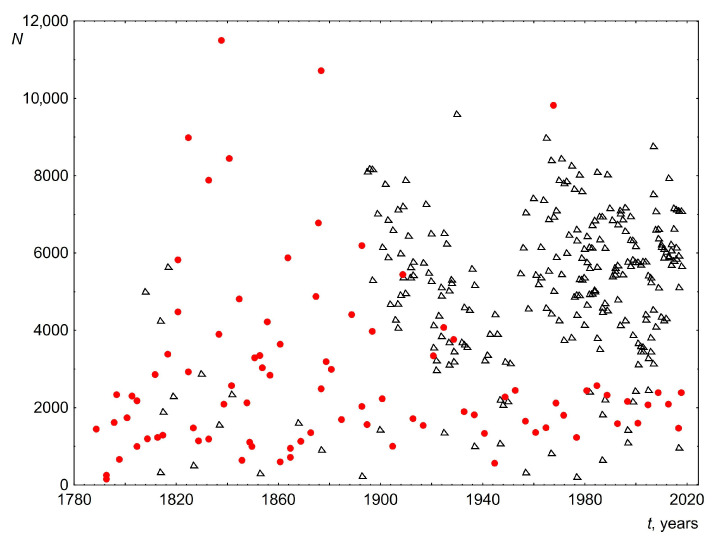
Number of words in the text N versus time t. Black triangles indicate data for the UK and red circles indicate data for the USA.

The average speech length in words was approximately 2800 for the USA and 5100 for the UK. As can be seen from Figure 2, in the vast majority of cases the number of words for each speech is more than 1000. This is a very good sample size for a reliable analysis. The difference in the average number of words for the USA and the UK is due to political tradition and historical reasons. This difference is not the subject of this article and does not affect the results presented below.

In further analysis, word length will be treated as a random variable. This term is used in the sense of mathematical statistics. One may wonder whether such an assumption can be used when the texts of political speeches are usually carefully prepared. The answer is yes, for two reasons. The first reason is that when preparing speeches, one does not consider the length of the words, but primarily their content and meaning. The second reason is that even if writers of political speeches paid special attention to the length of the words used (and, for example, considered the Menzerath–Altmann law or various readability indexes), many other controlled and uncontrolled factors, such as political, cultural, historical, psychological, etc., would have various multidirectional effects on the choice of words and therefore, from the point of view of mathematical statistics, word length would be random.

Statistical analysis was carried out in widespread professional commercial software MATLAB R2020 (The MathWorks). Distribution parameters were calculated using the Levenberg–Marquardt algorithm implemented in MATLAB. The commercial software Statistica 12.0 (TIBCO Software) was used to visualize the results and to validate and verify several calculations. The data (year of the speech and values corresponding to the processed word lengths of speeches) are open access [26].

## 3. Analysis of the Word Length Distribution Law

In the process of analyzing the law of distribution, 82 speeches by USA presidents and 245 speeches from the UK were used. Presumed multimodality excluded speeches from the following years: 1793, 1793, 1829, 1833, 1846, 1849, 2005 (for USA) and two speeches: 1814 and 1977 (for UK). Five continuous distributions containing no more than two parameters were used for analysis, namely the following: lognormal, Weibull, Rayleigh, folded normal and half normal (normal). Weibull and lognormal distributions are traditionally used in the study of word lengths, as discussed in the Introduction. Rayleigh distribution is very closely related to the Weibull distribution, and it was important to check whether word lengths are described by this simpler one-parameter distribution. Folded normal and half-normal distributions are directly related to the normal distribution, which is the most basic distribution in mathematical statistics. The belonging to the normal distribution is first of all checked before proceeding to more complex ones.

The ranking of these distributions by the quality of empirical data description was carried out according to the coefficient of determination: the closer the coefficient of determination was to 1, the higher the quality of distribution was (the highest is the first place, the lowest (worst) is the fifth place).

Table 1 and Table 2 summarize the calculation results presented in detail in Appendix A. From the tables we can see that the best distribution is the lognormal distribution. So, it took the first place for the overwhelming number of speeches (in 58 cases out of 82 for the USA and in 230 cases out of 245 for UK). Second place by a wide margin is the Weibull distribution, which was best in only 24 cases (out of 82) for the USA, and 15 cases (out of 245) for the UK. Note that the coefficients of determination for these distributions do not differ from each other remarkably but they differ statistically significantly. So, the average value of the coefficient of determination for the lognormal distribution was 0.998, and it was 0.995 for the Weibull distribution. For folded/half-normal and Rayleigh distributions, the coefficients of determination ranged from 0.979 to 0.986.

**Figure 3:**
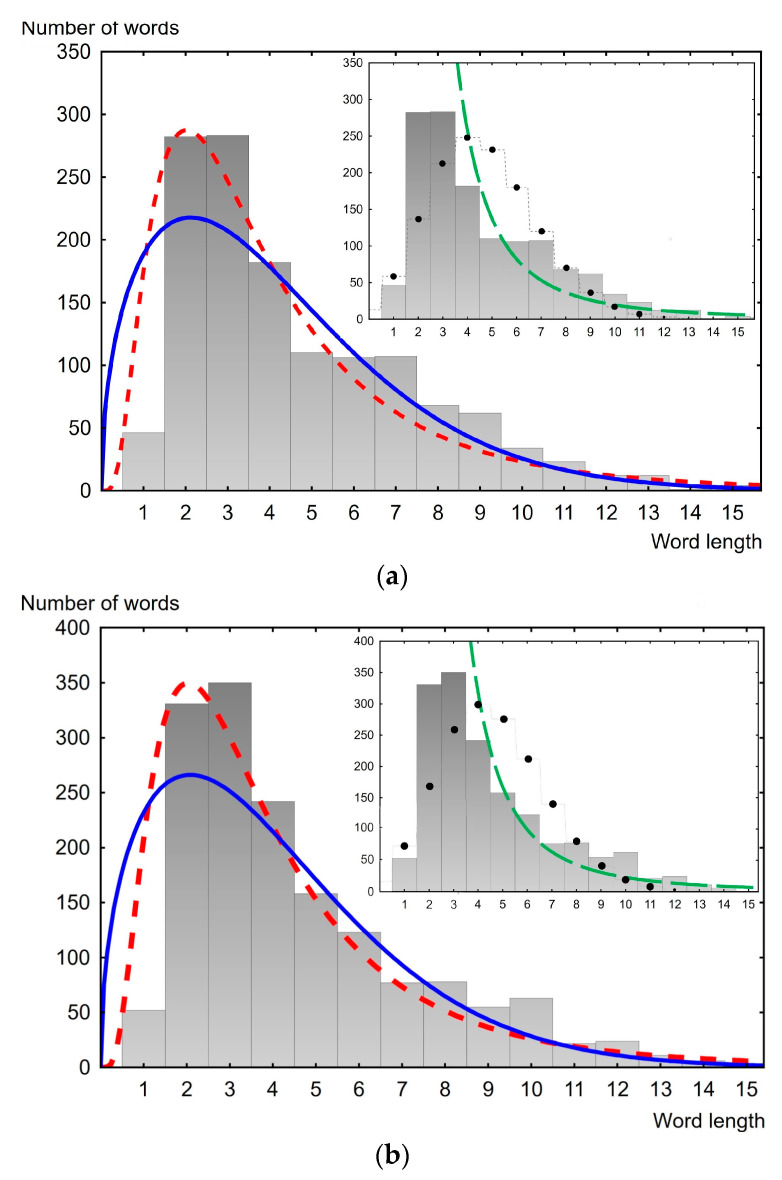
Word length distribution histogram. The dashed red line is the lognormal distribution, the solid blue line is the Weibull distribution. The inset shows the best possible approximation of the distribution histogram using a discrete Poisson distribution (black dots) and a power function of the form const/(word length), represented by the dashed green line. (**a**) USA speech, delivered in 1873, 1334 words. The determination coefficient is 0.995 for lognormal distribution (*µ* = 1.21, *σ* = 0.71); (**b**) UK speech, delivered in 1868; 1595 words. The determination coefficient is 0.998 for lognormal distribution (*µ* = 1.20, *σ* = 0.70).

**Figure 4:**
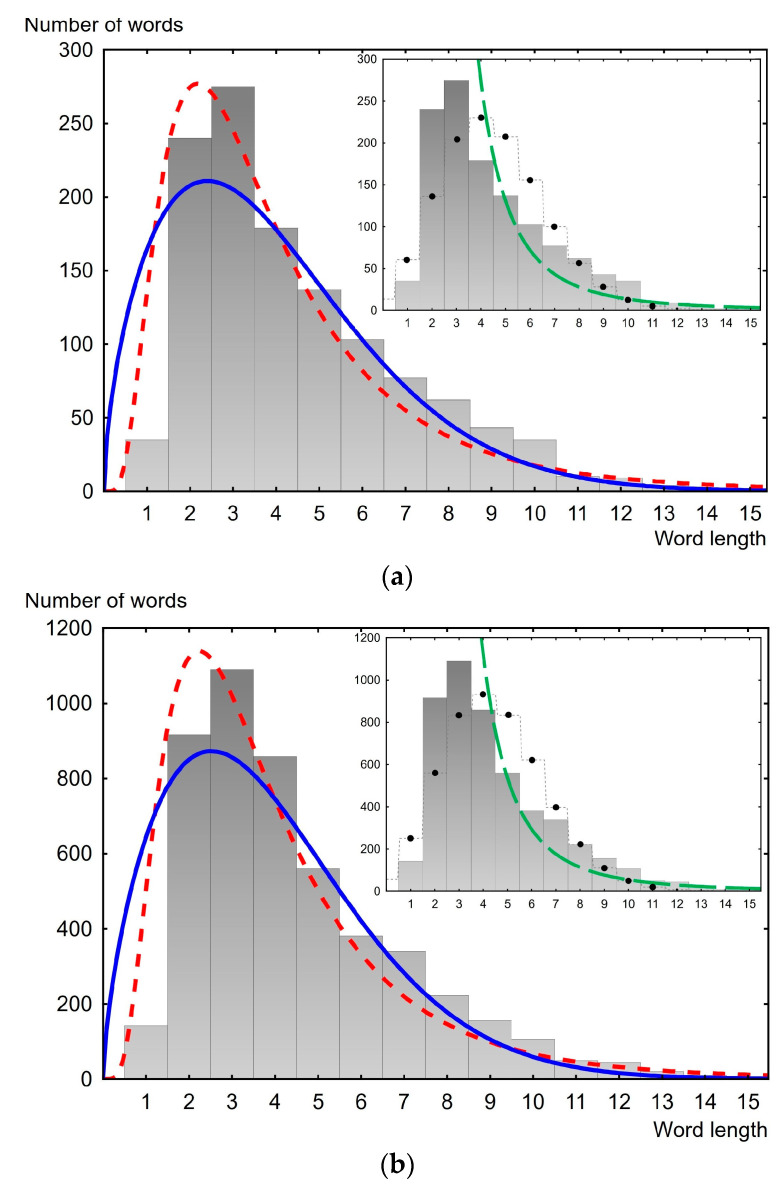
Word length distribution histogram. The dashed red line is the lognormal distribution, the solid blue line is the Weibull distribution. The inset shows the best possible approximation of the distribution histogram using a discrete Poisson distribution (black dots) and a power function of the form const/(word length), represented by the dashed green line. (**a**) USA speech, delivered in 1977, 1213 words. The coefficient of determination is 0.998 for lognormal distribution (*µ* = 1.20, *σ* = 0.65); (**b**) UK speech (Conservative party), delivered in 1978; 4898 words. The coefficient of determination is 0.999 for lognormal distribution (*µ* = 1.20, *σ* = 0.63).

Thus, based on the coefficient of determination, the lognormal distribution seems to be the most suitable for describing the law of distribution of word lengths in the texts of the considered public speeches. As is known, the lognormal distribution has the form $\frac{1}{2}+\frac{1}{2}erf\left[\frac{\ln\left(x\right)-µ}{\sigma \sqrt{2}}\right]$, where *σ* and *µ* are parameters. The computed parameters *σ* and *µ* for all analyzed speeches are presented in Appendix B. Examples of the description of empirical data of different years for the USA and the UK are presented in Figure 3 and Figure 4. As can be seen, the lognormal distribution better describes empirical distributions in comparison with its direct “competitor”, the Weibull distribution. This is most noticeable for small word lengths (lengths between one and four). At the same time, from Figure 3 and Figure 4, it can be seen that the lognormal distribution systematically underestimates the mode value of the empirical data by approximately one.

The insets in Figure 3 and Figure 4 show approximations of the empirical histograms using the discrete two-parameter Poisson function $\lambda^{k}e^{-\lambda }/k!$ and the one-parameter power function following the brevity law and Zipf’s law. As discussed in the Introduction, these functions are proposed to describe the distribution of word lengths. However, as can be seen from the presented data, these theoretical distributions (functions) are not applicable for the data we are studying.

Note that no significant differences were found in the parameters *µ* and *σ* of the lognormal distributions used to describe speeches delivered at approximately the same time by US presidents and members of various parliamentary parties in the UK.

## 4. Change in Word Length over Time

**Figure 5:**
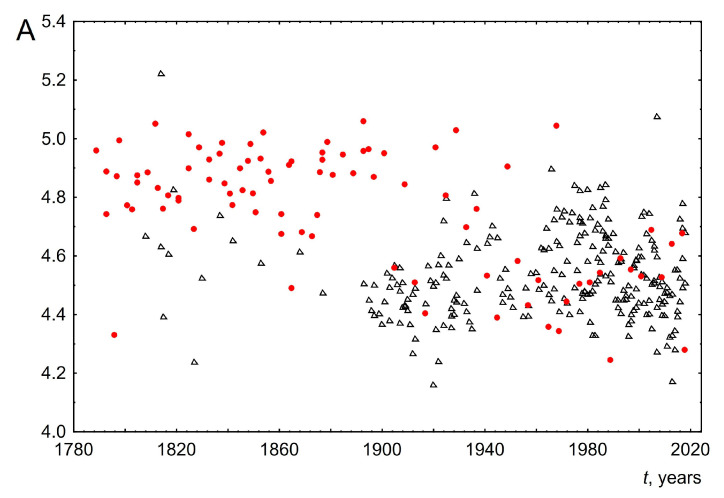
Average word length *A* as a function of the time speaking *t*. Red circles indicate data for the USA and black triangles indicate data for the UK.

**Figure 6:**
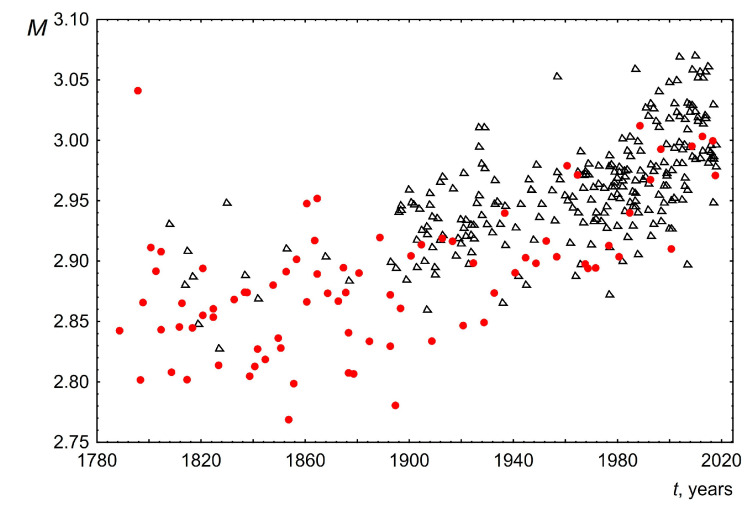
Mode word length *M* as a function of the time speaking *t*. Red circles indicate data for the USA and black triangles indicate data for the UK.

**Figure 7:**
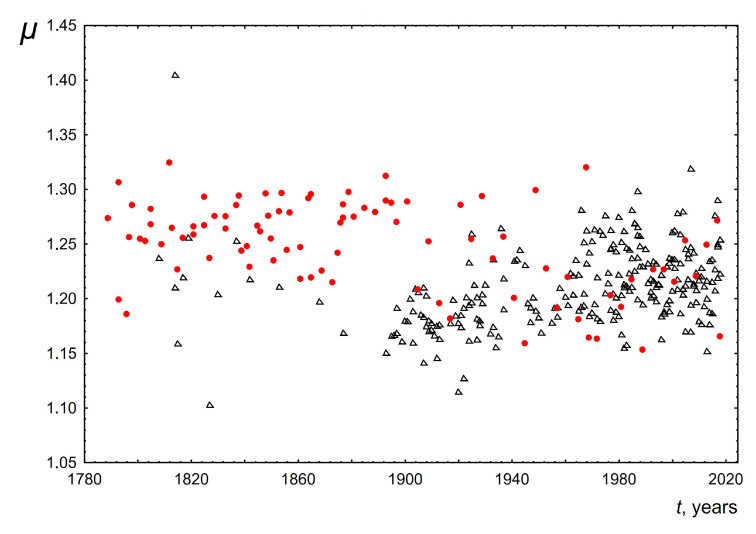
Behavior of the parameter *µ* of the lognormal distribution versus the time *t*. Red circles indicate data for the USA and black triangles indicate data for the UK.

**Figure 8:**
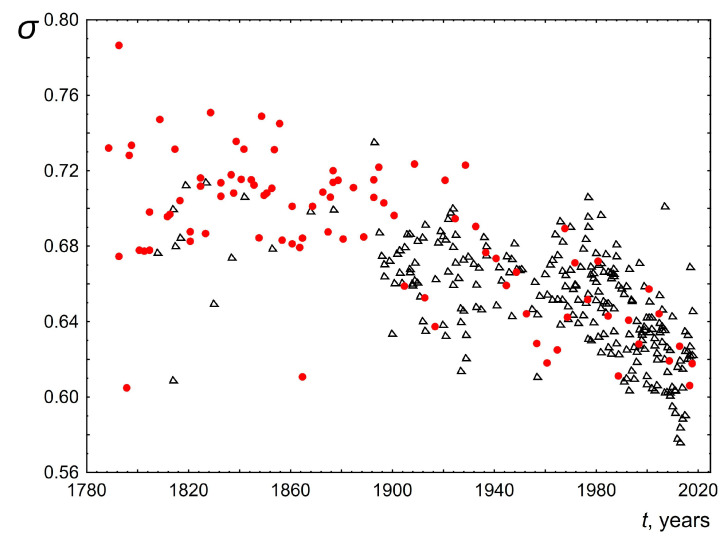
Behavior of the parameter *σ* of the lognormal distribution versus the time *t*. Red circles indicate data for the USA and black triangles indicate data for the UK.

A number of quantities were calculated to analyze the possible change in word length over time. They are listed below:

1. The average word length. To calculate this parameter, the total number of letters in a speech is divided by the number of words. The change in this parameter over time is shown in Figure 5. As can be seen from the graph, the average word length for UK speeches has remained almost unchanged over two hundred years and is about 4.5. For USA speeches, the result is more complicated. So, from 1789 to about 1950, the average length of words practically did not change and was about 4.9. However, then there is a small stepwise change in the average word length to 4.5. As a result, from 1950 to the present, the average length of words for both the USA and the UK is the same. Within quantitative linguistics it is impossible to understand the reasons for such behavior. However, based on historical facts, we will put forward the following hypotheses to explain this behavior. (1) Initially, USA presidents made speeches only before Congress; other citizens became acquainted with their speeches through newspapers. Such speeches only began to be fully broadcasted on radio and television after the Second World War. This may have led to the observed decrease in average word length. (2) The unchanged average word length for the UK can be explained, apparently, by the fact that the speeches were made before members of Parliament, a rather conservative representative body with strong centuries-old traditions. Adherence to tradition is a quality that is often used to describe British society as a whole. This is illustrated by the result obtained in the work about the unchanged average length of words of parliamentarians over more than two hundred years. (3) It was after the Second World War that a special relationship emerged between the USA and the UK. The term “special relationship” publicly emerged in Winston Churchill’s “Iron Curtain” speech of 1946. The special relationship is a term that is often used to describe the close historic, political, military, economic and cultural relations between USA and UK political leaders and elites. This could be the reason that the vocabulary of USA and UK politicians became very close after the war and, as a consequence, the average length of words is the same.

2. The median of the word length distribution for all speeches was found to be four and did not change over time.
3. The maximum word length of speeches delivered over 200 years also did not change and was in the range from 14 to 16. Examples of these most common words for the USA: accountability, administration, constitutional, accomplishment, irresponsibility. For the UK, these words are responsibility, congratulation, disestablishment, apprenticeship, discrimination, internationalism.
4. The mode for each of the speeches was calculated based on the averaging of the three most probable word lengths. The averaging was performed taking into account the probabilities of occurrence of these three values in the text. This was performed to make this parameter more sensitive to the shape and width of the length distribution near the maximum. The change in the mode of the word length distribution over time is shown in Figure 6. As can be seen, the mode of word lengths of public speeches for both the USA and the UK increases slightly over 200 years from 2.85 to 3.02. This change is approximately linear with a slope of 0.0005± 0.0002 for the USA and 0.0006 ± 0.0001 for the UK.
5. Figure 7 and Figure 8 show the dependence of the parameters of the lognormal distribution µ, σ on time. As can be seen, µ does not depend on time, remaining approximately equal to 1.25. At the same time, the parameter σ slightly decreases over 200 years from 0.74 to 0.62. These results are consistent with the results presented in Figure 5 and Figure 6. Indeed, as we know, the mean value for a lognormal distribution is related to *µ, σ* as $exp(µ+\sigma^{2}/2)$. As a consequence, when µ is constant and σ decreases the mean value will decrease. On the other hand, since the mode is related to *µ*, *σ* as $exp(µ-\sigma^{2})$, then when µ remains constant and σ decreases, the mode should increase. The above agreement in the behavior of the parameters of the lognormal distribution µ, σ (Figure 7 and Figure 8) with the results based on the analysis of directly empirical histograms (Figure 5 and Figure 6) provides an additional argument for the applicability of the lognormal distribution to describe the distribution of word lengths.

**Figure 9:**
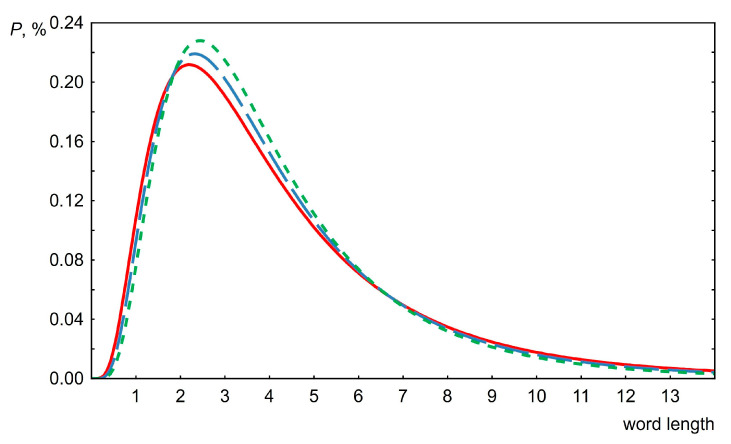
Lognormal distribution showing the change in word length distributions over time. Data for the USA, the *µ* parameter is 1.25, the *σ* parameters are 0.68 (red line), 0.64 (blue line) and 0.60 (green line) for 1815, 1915 and 2015, respectively.

The change in the probability density of the lognormal distribution with time is presented in Figure 9. It can be seen that this change is very insignificant over two hundred years.

## 5. Conclusions

This paper shows that the lognormal distribution is better applicable to describe the distribution of word lengths in the texts of public speeches by politicians. This is significantly different from the result obtained for the sentence length distribution, in which the Weibull distribution is preferred [9]. As a consequence, we can conclude that the principle of least effort does not have a significant impact on the length of words used by politicians. As is known, an important reason for the appearance of a lognormal distribution is the presence of a multiplicative random process that determines the random variable [27]. In our case, such a random variable is the length of the word.

It is found that word length in public speeches of USA and UK politicians has remained almost unchanged over the last two hundred years. There has been a very slight decrease in the average value for the USA and a slight increase in the most probable word length (mode). This result differs significantly from the results obtained previously for sentence lengths, the lengths of which decreased significantly over time [9]. The result obtained for words is quite surprising. Indeed, for such a long period of time, many very significant historical events have occurred for humanity, new techniques and technologies have appeared and improved explosively and enormous cultural and social changes have taken place in society. All this inevitably led to the emergence of new words and should have affected the frequency of use of existing words. However, as statistical analysis has shown, the length of words used has not changed much. Apparently, the length of words is statistically a very conservative parameter, which turns out to be insensitive to the above changes. These changes affect words in different ways, which do not affect their average length.

The results obtained in this paper question not only the applicability of the principle of least effort, but indicate a possible limitation of the Menzerath–Altmann, Zipf and brevity laws for analyzing word lengths in public speaking. We base this conclusion on the following logic. (1) As discussed above (see also [9]) the feasibility of the principle of least effort should lead to a Weibull distribution for word lengths and to a decrease in average word lengths over time. However, neither one nor the other was found. (2) Since it was previously discovered in [9] that the length of sentences decreases significantly, then, based on the constancy of the length of words over time obtained for the same speeches, it follows that the Menzerath–Altmann law is inapplicable for the sentence/word pair in the speeches of politicians. (3) Since the consequence of the Zipf and brevity laws is a power–law dependence of the frequency of words on their length, the established lognormal distribution for word lengths shows the limitations of these laws, at least when considering all word lengths, including the shortest ones.

There are quite a lot of laws in quantitative linguistics, only a small part of which we critically discuss in this article. These laws (statements) do not have the same degree of generality and universal acceptance as, for example, can be seen in physics. If we use the terminology of physics, then these statements in quantitative linguistics can be called working hypotheses rather than laws. These working hypotheses need clarification and adjustment. Thanks to research like the present one, this work of turning working hypotheses into laws is taking place.

It is important to emphasize in conclusion, following [4], that word length is an essential property of a word; from the point of view of quantitative linguistics, word length with its relationships with other linguistic structures and levels provides information that is an important part of the general theory of language.

## Figures and Tables

**Table 1:** Ranking of distributions according to the coefficient of determination criterion. USA speeches. The total number is 82.

Place	Lognormal	Weibull	Folded Normal	Rayleigh	Half Normal
1	58	24	0	0	0
2	24	58	0	0	0
3	0	0	67	15	0
4	0	0	15	9	58
5	0	0	0	14	68
6	0	0	0	44	38

**Table 2:** Ranking of distributions according to the coefficient of determination criterion. UK speeches. The total number is 245.

Place	Lognormal	Weibull	Folded Normal	Rayleigh	Half Normal
1	230	15	0	0	0
2	14	230	1	0	0
3	0	0	145	99	1
4	0	0	99	83	63
5	0	0	0	37	208
6	1	0	0	26	218

## Data Availability

The data presented in this study are available on request from the corresponding author.

## Appendix A

Ranking the suitability of theoretical distribution laws for the observed empirical word length distributions based on the determination coefficient (R^2^) criterion. After the name of the distribution, its R^2^ is given:

**Table A1:** Data for the USA (distribution laws).

Year, Party	I Place, R^2^	II Place, R^2^	III Place, R^2^
1789	Lognormal, 0.995	Weibull, 0.993	Folded normal, 0.987
1796	Lognormal, 0.998	Weibull, 0.996	Rayleigh, 0.992
1797	Weibull, 0.993	Lognormal, 0.992	Folded normal, 0.989
1798	Lognormal, 0.989	Weibull, 0.988	Folded normal, 0.981
1801	Lognormal, 0.997	Weibull, 0.994	Folded normal, 0.985
1803	Lognormal, 0.996	Weibull, 0.994	Folded normal 0.985
1805	Lognormal, 0.996	Weibull, 0.995	Folded normal, 0.988
1805	Weibull, 0.994	Lognormal, 0.994	Folded normal, 0.988
1809	Lognormal, 0.993	Weibull, 0.992	Folded normal, 0.987
1812	Weibull, 0.996	Lognormal, 0.992	Folded normal, 0.991
1813	Lognormal, 0.995	Weibull, 0.994	Folded normal, 0.987
1815	Lognormal, 0.993	Weibull, 0.992	Folded normal, 0.986
1817	Lognormal, 0.994	Weibull, 0.994	Folded normal, 0.987
1821	Lognormal, 0.996	Weibull, 0.995	Folded normal, 0.987
1821	Lognormal, 0.994	Weibull, 0.994	Folded normal, 0.986
1825	Lognormal, 0.992	Weibull, 0.992	Folded normal, 0.986
1825	Lognormal, 0.993	Weibull, 0.991	Folded normal, 0.984
1827	Lognormal, 0.994	Weibull, 0.992	Folded normal, 0.984
1833	Lognormal, 0.993	Weibull, 0.992	Folded normal, 0.985
1837	Weibull, 0.994	Lognormal, 0.991	Folded normal, 0.989
1838	Weibull, 0.993	Lognormal, 0.992	Folded normal, 0.988
1839	Lognormal, 0.993	Weibull 0.992	Folded normal, 0.987
1841	Lognormal, 0.993	Weibull, 0.993	Folded normal, 0.987
1842	Lognormal, 0.993	Weibull, 0.993	Folded normal, 0.987
1845	Lognormal, 0.993	Weibull, 0.993	Folded normal, 0.987
1848	Weibull, 0.995	Lognormal, 0.993	Folded normal, 0.99
1850	Lognormal, 0.993	Weibull, 0.993	Folded normal, 0.986
1851	Lognormal, 0.993	Weibull, 0.992	Folded normal, 0.985
1853	Lognormal, 0.994	Weibull, 0.994	Folded normal, 0.988
1854	Weibull, 0.991	Lognormal, 0.988	Folded normal, 0.986
1856	Lognormal, 0.99	Weibull, 0.988	Folded normal, 0.982
1857	Weibull, 0.995	Lognormal, 0.995	Folded normal, 0.989
1861	Lognormal, 0.996	Weibull, 0.994	Folded normal, 0.986
1861	Lognormal, 0.997	Weibull, 0.993	Folded normal, 0.983
1864	Lognormal, 0.994	Weibull, 0.994	Folded normal, 0.987
1865	Lognormal, 0.998	Weibull, 0.993	Rayleigh, 0.989
1865	Lognormal, 0.992	Weibull, 0.99	Folded normal, 0.982
1868	Weibull, 0.994	Lognormal, 0.992	Folded normal, 0.989
1869	Weibull, 0.995	Lognormal, 0.994	Folded normal, 0.99
1873	Lognormal, 0.995	Weibull, 0.994	Folded normal, 0.987
1875	Lognormal, 0.996	Weibull, 0.994	Folded normal 0.986
1876	Lognormal, 0.994	Weibull, 0.994	Folded normal, 0.987
1877	Weibull, 0.993	Lognormal, 0.992	Folded normal, 0.989
1877	Weibull, 0.992	Lognormal, 0.991	Folded normal, 0.987
1879	Weibull, 0.992	Lognormal, 0.989	Folded normal, 0.986
1881	Lognormal, 0.995	Weibull, 0.992	Folded normal, 0.984
1885	Lognormal, 0.992	Weibull, 0.992	Folded normal, 0.985
1889	Lognormal, 0.995	Weibull, 0.994	Folded normal, 0.986
1893	Weibull, 0.993	Lognormal, 0.991	Folded normal, 0.988
1893	Weibull, 0.993	Lognormal, 0.992	Folded normal, 0.987
1895	Weibull, 0.992	Lognormal, 0.99	Folded normal, 0.987
1897	Lognormal, 0.992	Weibull, 0.992	Folded normal, 0.986
1901	Lognormal, 0.993	Weibull, 0.992	Folded normal, 0.984
1905	Lognormal, 0.998	Weibull, 0.994	Folded normal, 0.985
1909	Weibull, 0.993	Lognormal, 0.992	Folded normal, 0.987
1913	Lognormal, 0.998	Weibull, 0.994	Rayleigh, 0.984
1917	Lognormal, 0.998	Weibull, 0.994	Rayleigh, 0.987
1921	Lognormal, 0.993	Weibull, 0.993	Folded normal, 0.987
1925	Lognormal, 0.995	Weibull, 0.993	Folded normal, 0.986
1929	Weibull, 0.993	Lognormal, 0.993	Folded normal, 0.987
1933	Weibull, 0.996	Lognormal, 0.996	Folded normal, 0.989
1937	Lognormal, 0.996	Weibull, 0.996	Folded normal, 0.989
1941	Lognormal, 0.997	Weibull, 0.993	Folded normal, 0.984
1945	Lognormal, 0.999	Weibull, 0.995	Folded normal, 0.986
1949	Weibull, 0.996	Lognormal, 0.995	Folded normal, 0.991
1953	Lognormal, 0.997	Weibull, 0.995	Folded normal, 0.987
1957	Lognormal, 0.998	Weibull, 0.995	Rayleigh, 0.99
1961	Lognormal, 0.998	Weibull, 0.996	Rayleigh, 0.992
1965	Lognormal, 0.997	Weibull, 0.996	Rayleigh, 0.99
1969	Lognormal, 0.998	Weibull, 0.996	Rayleigh, 0.988
1973	Lognormal, 0.999	Weibull, 0.993	Folded normal, 0.983
1977	Lognormal, 0.998	Weibull, 0.996	Folded normal, 0.988
1981	Lognormal, 0.997	Weibull, 0.995	Folded normal, 0.987
1985	Lognormal, 0.998	Weibull, 0.996	Folded normal, 0.988
1989	Lognormal, 0.999	Weibull, 0.996	Rayleigh, 0.993
1993	Lognormal, 0.997	Weibull, 0.994	Rayleigh, 0.986
1997	Lognormal, 0.998	Weibull, 0.995	Rayleigh, 0.989
2001	Weibull, 0.996	Lognormal, 0.995	Folded normal, 0.99
2009	Lognormal, 0.998	Weibull, 0.995	Rayleigh, 0.99
2013	Lognormal, 0.998	Weibull, 0.996	Rayleigh, 0.99
2017	Lognormal, 0.997	Weibull, 0.995	Rayleigh, 0.992
2021	Lognormal, 0.998	Weibull, 0.996	Rayleigh, 0.992

**Table A2:** Data for UK (distribution laws).

Year, Party	I Place, R^2^	II Place, R^2^	III Place, R^2^
1808	Lognormal, 0.996	Weibull, 0.996	Folded normal, 0.989
1814	Lognormal, 0.996	Weibull, 0.993	Folded normal, 0.985
1815	Lognormal, 0.998	Weibull, 0.996	Folded normal, 0.988
1817	Lognormal, 0.995	Weibull, 0.995	Folded normal, 0.987
1819	Lognormal, 0.993	Weibull, 0.993	Folded normal, 0.986
1827	Lognormal, 0.996	Weibull, 0.995	Folded normal, 0.987
1830	Lognormal, 0.998	Weibull, 0.995	Rayleigh, 0.987
1837	Lognormal, 0.996	Weibull, 0.994	Folded normal, 0.985
1842	Weibull, 0.995	Lognormal, 0.994	Folded normal, 0.99
1853	Lognormal, 0.995	Weibull, 0.994	Folded normal, 0.985
1868	Lognormal, 0.998	Weibull, 0.993	Folded normal, 0.985
1877	Lognormal, 0.996	Weibull, 0.992	Folded normal, 0.984
1893	Lognormal, 0.993	Weibull, 0.987	Half Normal, 0.979
1895 Liberal	Lognormal, 0.998	Weibull, 0.995	Folded normal, 0.986
1896 Liberal	Lognormal, 0.998	Weibull, 0.995	Folded normal, 0.986
1897 Conservative	Lognormal, 0.998	Weibull, 0.995	Folded normal, 0.986
1897 Liberal	Lognormal, 0.998	Weibull, 0.995	Folded normal, 0.986
1899 Liberal	Lognormal, 0.998	Weibull, 0.993	Folded normal, 0.984
1900 Conservative	Lognormal, 0.998	Weibull, 0.996	Rayleigh, 0.988
1901 Liberal	Lognormal, 0.998	Weibull, 0.994	Folded normal, 0.984
1902 Conservative	Lognormal, 0.997	Weibull, 0.996	Folded normal, 0.988
1903 Conservative	Lognormal, 0.998	Weibull, 0.997	Folded normal, 0.99
1903 Liberal	Lognormal, 0.998	Weibull, 0.994	Folded normal, 0.984
1904 Conservative	Lognormal, 0.997	Weibull, 0.996	Folded normal, 0.989
1905 Liberal	Lognormal, 0.997	Weibull, 0.995	Folded normal, 0.987
1906 Conservative	Lognormal, 0.997	Weibull, 0.994	Folded normal, 0.987
1907	Lognormal, 0.998	Weibull 0.994	Folded normal, 0.986
1907 Conservative	Lognormal, 0.998	Weibull, 0.996	Folded normal, 0.988
1907 Liberal	Lognormal, 0.998	Weibull, 0.995	Folded normal, 0.986
1908 Conservative	Lognormal, 0.998	Weibull, 0.995	Folded normal, 0.986
1908 Liberal	Lognormal, 0.998	Weibull, 0.995	Folded normal, 0.986
1909 Conservative	Lognormal, 0.998	Weibull, 0.996	Folded normal, 0.988
1909 Liberal	Lognormal, 0.998	Weibull, 0.994	Folded normal, 0.985
1910 Conservative	Lognormal, 0.998	Weibull, 0.996	Folded normal, 0.989
1910 Liberal	Lognormal, 0.998	Weibull, 0.995	Folded normal, 0.987
1911 Conservative	Lognormal, 0.998	Weibull, 0.996	Folded normal, 0.988
1912 Conservative	Lognormal, 0.999	Weibull, 0.996	Rayleigh, 0.989
1912 Liberal	Lognormal, 0.997	Weibull, 0.995	Folded normal, 0.987
1913 Conservative	Lognormal, 0.999	Weibull, 0.996	Rayleigh, 0.989
1913 Liberal	Lognormal, 0.997	Weibull, 0.994	Folded normal, 0.986
1917	Lognormal 0.999	Weibull, 0.996	Folded normal, 0.988
1918 Liberal	Lognormal, 0.998	Weibull, 0.994	Folded normal, 0.986
1919 Liberal	Lognormal, 0.997	Weibull, 0.995	Folded normal, 0.987
1920 Conservative	Lognormal, 0.999	Weibull, 0.995	Rayleigh, 0.988
1920 Liberal	Lognormal, 0.997	Weibull, 0.993	Folded normal, 0.985
1921 Conservative	Lognormal, 0.999	Weibull, 0.997	Rayleigh, 0.99
1921 Liberal	Lognormal, 0.998	Weibull, 0.995	Folded normal, 0.986
1922 Conservative	Lognormal, 0.998	Weibull, 0.996	Folded normal, 0.989
1922 Liberal	Lognormal, 0.997	Weibull, 0.993	Folded normal, 0.984
1923 Liberal	Lognormal, 0.996	Weibull, 0.993	Folded normal, 0.984
1924 Conservative	Lognormal, 0.998	Weibull, 0.995	Folded normal, 0.987
1924 Labour	Lognormal, 0.997	Weibull, 0.993	Folded normal, 0.984
1924 Liberal	Lognormal, 0.995	Weibull, 0.993	Folded normal, 0.985
1925 Conservative	Lognormal, 0.997	Weibull, 0.994	Folded normal, 0.985
1925 Liberal	Lognormal, 0.995	Weibull, 0.993	Folded normal, 0.985
1926 Conservative	Lognormal, 0.998	Weibull, 0.995	Folded normal, 0.986
1927	Lognormal, 0.999	Weibull, 0.996	Rayleigh, 0.988
1927 Conservative	Lognormal, 0.998	Weibull, 0.996	Rayleigh, 0.988
1927 Liberal	Lognormal, 0.999	Weibull, 0.996	Rayleigh, 0.991
1928 Conservative	Lognormal, 0.998	Weibull, 0.996	Folded normal, 0.988
1928 Liberal	Lognormal, 0.998	Weibull, 0.994	Folded normal, 0.984
1929 Conservative	Lognormal, 0.998	Weibull, 0.995	Rayleigh, 0.988
1929 Liberal	Lognormal, 0.999	Weibull, 0.994	Rayleigh, 0.989
1930 Liberal	Lognormal, 0.997	Weibull, 0.995	Folded normal, 0.987
1932 Conservative	Lognormal, 0.998	Weibull, 0.993	Folded normal, 0.984
1932 Liberal	Lognormal, 0.997	Weibull, 0.995	Rayleigh, 0.984
1933 Conservative	Lognormal, 0.999	Weibull, 0.993	Rayleigh, 0.985
1934 Conservative	Lognormal, 0.998	Weibull, 0.993	Folded normal, 0.983
1935 Conservative	Lognormal, 0.998	Weibull, 0.996	Folded normal, 0.988
1936 Liberal	Lognormal, 0.994	Weibull, 0.993	Folded normal, 0.986
1937	Lognormal, 0.997	Weibull, 0.996	Folded normal, 0.989
1937 Liberal	Lognormal, 0.996	Weibull, 0.994	Folded normal, 0.985
1941 Liberal	Lognormal, 0.998	Weibull, 0.994	Rayleigh, 0.985
1942 Liberal	Lognormal, 0.996	Weibull, 0.995	Folded normal, 0.987
1943 Liberal	Lognormal, 0.997	Weibull, 0.994	Folded normal, 0.986
1945 Liberal	Lognormal, 0.996	Weibull, 0.994	Folded normal, 0.986
1946 Labour	Lognormal, 0.998	Weibull, 0.994	Folded normal, 0.984
1947	Lognormal 0.998	Weibull, 0.996	Folded normal, 0.988
1947 Labour	Lognormal, 0.998	Weibull, 0.993	Folded normal, 0.984
1948 Labour	Lognormal, 0.997	Weibull, 0.995	Folded normal, 0.986
1949 Labour	Lognormal, 0.998	Weibull, 0.994	Folded normal, 0.985
1950 Labour	Lognormal, 0.998	Weibull, 0.995	Folded normal, 0.985
1951 Labour	Lognormal, 0.998	Weibull, 0.994	Folded normal, 0.985
1955 Conservative	Lognormal, 0.998	Weibull, 0.995	Folded normal, 0.987
1956 Conservative	Lognormal, 0.998	Weibull, 0.994	Folded normal, 0.985
1957	Lognormal 0.999	Weibull, 0.994	Rayleigh, 0.99
1957 Conservative	Lognormal, 0.998	Weibull, 0.994	Rayleigh, 0.986
1958 Conservative	Lognormal, 0.998	Weibull, 0.995	Folded normal, 0.986
1960 Conservative	Lognormal, 0.998	Weibull, 0.993	Folded normal, 0.983
1961 Conservative	Lognormal, 0.998	Weibull, 0.995	Folded normal, 0.986
1962 Conservative	Lognormal, 0.997	Weibull, 0.994	Folded normal, 0.985
1963 Conservative	Lognormal, 0.997	Weibull, 0.994	Folded normal, 0.985
1963 Liberal	Lognormal, 0.998	Weibull, 0.996	Folded normal, 0.987
1964 Labour	Lognormal, 0.996	Weibull, 0.995	Folded normal, 0.989
1965 Conservative	Lognormal, 0.999	Weibull, 0.995	Folded normal, 0.986
1965 Labour	Lognormal, 0.997	Weibull, 0.995	Folded normal, 0.988
1966 Conservative	Lognormal, 0.998	Weibull, 0.994	Rayleigh, 0.986
1966 Labour	Weibull, 0.995	Lognormal, 0.994	Folded normal, 0.988
1967	Lognormal, 0.997	Weibull, 0.995	Folded normal, 0.988
1967 Conservative	Lognormal, 0.998	Weibull, 0.994	Rayleigh, 0.984
1967 Labour	Lognormal, 0.997	Weibull, 0.995	Folded normal, 0.986
1968 Conservative	Lognormal, 0.998	Weibull, 0.994	Folded normal, 0.985
1968 Labour	Lognormal, 0.997	Weibull, 0.996	Folded normal, 0.988
1969 Conservative	Lognormal, 0.998	Weibull, 0.996	Rayleigh, 0.988
1969 Labour	Lognormal, 0.997	Weibull, 0.996	Folded normal, 0.989
1970 Conservative	Lognormal, 0.997	Weibull, 0.991	Folded normal, 0.981
1970 Labour	Lognormal, 0.997	Weibull, 0.995	Folded normal, 0.987
1971 Conservative	Lognormal, 0.998	Weibull, 0.995	Rayleigh, 0.987
1971 Labour	Lognormal, 0.998	Weibull, 0.996	Folded normal, 0.989
1972 Conservative	Lognormal, 0.998	Weibull, 0.995	Folded normal, 0.986
1972 Labour	Lognormal, 0.998	Weibull, 0.996	Folded normal, 0.988
1973 Conservative	Lognormal, 0.998	Weibull, 0.994	Rayleigh, 0.986
1973 Labour	Lognormal, 0.997	Weibull, 0.997	Folded normal, 0.99
1974 Labour	Lognormal, 0.996	Weibull, 0.995	Folded normal, 0.988
1975 Conservative	Lognormal, 0.998	Weibull, 0.995	Rayleigh, 0.986
1975 Labour	Lognormal, 0.996	Weibull, 0.995	Folded normal, 0.987
1976 Conservative	Lognormal, 0.998	Weibull, 0.994	Folded normal, 0.985
1976 Labour	Lognormal, 0.997	Weibull, 0.996	Folded normal, 0.988
1977 Conservative	Lognormal, 0.998	Weibull, 0.995	Rayleigh, 0.988
1977 Liberal a	Weibull, 0.995	Lognormal, 0.995	Folded normal, 0.989
1977 Liberal b	Weibull, 0.995	Lognormal, 0.995	Folded normal, 0.989
1978 Conservative	Lognormal, 0.999	Weibull, 0.996	Rayleigh, 0.989
1978 Labour	Lognormal, 0.998	Weibull, 0.995	Folded normal, 0.986
1978 Liberal	Lognormal, 0.997	Weibull, 0.996	Folded normal, 0.989
1979 Conservative	Lognormal, 0.998	Weibull, 0.995	Rayleigh, 0.987
1979 Labour	Lognormal, 0.998	Weibull, 0.995	Folded normal, 0.985
1979 Liberal	Lognormal, 0.997	Weibull, 0.995	Folded normal, 0.987
1980 Conservative	Lognormal, 0.998	Weibull, 0.996	Folded normal, 0.988
1980 Labour	Lognormal, 0.998	Weibull, 0.994	Folded normal, 0.985
1980 Liberal	Weibull, 0.996	Lognormal, 0.995	Folded normal, 0.989
1981 Conservative	Lognormal, 0.998	Weibull, 0.996	Rayleigh, 0.989
1981 Labour	Lognormal, 0.998	Weibull, 0.995	Folded normal, 0.987
1981 Liberal	Lognormal, 0.997	Weibull, 0.995	Folded normal, 0.986
1982 Conservative	Lognormal, 0.998	Weibull, 0.996	Rayleigh, 0.99
1982 Labour	Lognormal, 0.999	Weibull, 0.994	Rayleigh, 0.986
1982 Liberal	Lognormal, 0.996	Weibull, 0.995	Folded normal, 0.988
1982 SDP-Liberal Alliance	Lognormal, 0.995	Weibull, 0.993	Folded normal, 0.985
1983 Conservative	Lognormal, 0.998	Weibull, 0.995	Rayleigh, 0.988
1983 Labour	Lognormal, 0.998	Weibull, 0.995	Folded normal, 0.987
1983 Liberal	Lognormal, 0.996	Weibull, 0.996	Folded normal, 0.988
1984 Conservative	Lognormal, 0.998	Weibull, 0.995	Rayleigh, 0.987
1984 Labour	Lognormal, 0.997	Weibull, 0.993	Folded normal, 0.983
1984 Liberal	Lognormal, 0.996	Weibull, 0.995	Folded normal, 0.988
1985 Conservative	Lognormal, 0.998	Weibull, 0.996	Rayleigh, 0.989
1985 Labour	Lognormal, 0.998	Weibull, 0.995	Folded normal, 0.986
1985 Liberal	Lognormal, 0.996	Weibull, 0.996	Folded normal, 0.988
1986 Conservative	Weibull, 0.997	Lognormal, 0.997	Rayleigh, 0.992
1986 Labour	Lognormal, 0.997	Weibull, 0.994	Folded normal, 0.986
1986 Liberal	Weibull, 0.996	Lognormal, 0.996	Folded normal, 0.99
1987 Conservative	Lognormal, 0.997	Weibull, 0.997	Rayleigh, 0.989
1987	Weibull 0.997	Lognormal, 0.996	Folded normal 0.991
1987 Labour	Lognormal, 0.998	Weibull, 0.993	Folded normal, 0.984
1987 SDP-Liberal Alliance a	Lognormal, 0.997	Weibull, 0.994	Folded normal, 0.985
1987 SDP-Liberal Alliance b	Lognormal, 0.997	Weibull, 0.995	Folded normal, 0.987
1988 Conservative	Lognormal, 0.997	Weibull, 0.996	Rayleigh, 0.989
1988 Labour	Lognormal, 0.996	Weibull, 0.994	Folded normal, 0.987
1988 Liberal	Weibull, 0.996	Lognormal, 0.996	Folded normal, 0.99
1989 Conservative	Lognormal, 0.998	Weibull, 0.996	Rayleigh, 0.991
1989 Labour	Lognormal, 0.998	Weibull, 0.993	Folded normal, 0.983
1990 Labour	Lognormal, 0.996	Weibull, 0.994	Folded normal, 0.986
1991 Conservative	Lognormal, 0.999	Weibull, 0.997	Rayleigh, 0.993
1991 Labour	Lognormal, 0.996	Weibull, 0.995	Folded normal, 0.987
1992 Conservative	Lognormal, 0.999	Weibull, 0.996	Rayleigh, 0.992
1992 Labour	Lognormal, 0.997	Weibull, 0.995	Folded normal, 0.988
1992 Liberal Democrat	Lognormal, 0.999	Weibull, 0.996	Rayleigh, 0.99
1993 Conservative	Lognormal, 0.999	Weibull, 0.997	Rayleigh, 0.993
1993 Labour	Lognormal, 0.998	Weibull, 0.996	Rayleigh, 0.989
1993 Liberal Democrat	Lognormal, 0.998	Weibull, 0.996	Folded normal, 0.988
1994 Conservative	Lognormal, 0.998	Weibull, 0.997	Rayleigh, 0.992
1994 Labour	Lognormal, 0.998	Weibull, 0.996	Folded normal, 0.988
1994 Liberal Democrat	Lognormal, 0.998	Weibull, 0.996	Folded normal, 0.988
1995 Conservative	Lognormal, 0.999	Weibull, 0.997	Rayleigh, 0.993
1995 Labour	Weibull, 0.998	Lognormal, 0.997	Rayleigh, 0.992
1996 Conservative	Lognormal, 0.998	Weibull, 0.997	Rayleigh, 0.991
1996 Labour	Lognormal, 0.999	Weibull, 0.996	Rayleigh, 0.989
1996 Liberal Democrat	Lognormal, 0.998	Weibull, 0.996	Rayleigh, 0.991
1997 Conservative	Lognormal, 0.998	Weibull, 0.997	Rayleigh, 0.991
1997	Lognormal, 0.996	Weibull, 0.994	Folded normal 0.986
1997 Labour	Lognormal, 0.998	Weibull, 0.996	Folded normal, 0.988
1997 Labour	Lognormal, 0.998	Weibull, 0.997	Rayleigh, 0.99
1998 Conservative	Lognormal, 0.998	Weibull, 0.996	Rayleigh, 0.99
1998 Labour	Lognormal, 0.997	Weibull, 0.997	Rayleigh, 0.99
1998 Liberal Democrat	Lognormal, 0.998	Weibull, 0.996	Rayleigh, 0.99
1999 Conservative	Lognormal, 0.998	Weibull, 0.996	Rayleigh, 0.989
1999 Labour	Lognormal, 0.998	Weibull, 0.996	Rayleigh, 0.99
1999 Liberal Democrat a	Lognormal, 0.999	Weibull, 0.994	Folded normal, 0.984
1999 Liberal Democrat b	Lognormal, 0.997	Weibull, 0.996	Rayleigh, 0.989
2000 Conservative	Lognormal, 0.999	Weibull, 0.995	Rayleigh, 0.992
2000 Labour	Lognormal, 0.997	Weibull, 0.997	Folded normal, 0.99
2000 Liberal Democrat	Lognormal, 0.998	Weibull, 0.996	Rayleigh, 0.992
2001 Conservative	Lognormal, 0.997	Weibull, 0.997	Folded normal, 0.989
2001 Labour	Lognormal, 0.997	Weibull, 0.997	Folded normal, 0.99
2001 Liberal Democrat	Lognormal, 0.997	Weibull, 0.996	Folded normal, 0.987
2002 Conservative	Lognormal, 0.999	Weibull, 0.996	Rayleigh, 0.993
2002 Labour	Lognormal, 0.998	Weibull, 0.996	Folded normal, 0.988
2002 Liberal Democrat	Lognormal, 0.997	Weibull, 0.997	Folded normal, 0.989
2003 Conservative	Lognormal, 0.999	Weibull, 0.996	Rayleigh, 0.992
2003 Labour	Lognormal, 0.998	Weibull, 0.995	Rayleigh, 0.988
2003 Liberal Democrat	Lognormal, 0.998	Weibull, 0.996	Rayleigh, 0.99
2004 Conservative	Lognormal, 0.999	Weibull, 0.997	Rayleigh, 0.993
2004 Labour	Lognormal, 0.998	Weibull, 0.996	Rayleigh, 0.991
2004 Liberal Democratl	Lognormal, 0.998	Weibull, 0.996	Rayleigh, 0.991
2005 Conservative	Lognormal, 0.999	Weibull, 0.995	Rayleigh, 0.989
2005 Labour	Lognormal, 0.998	Weibull, 0.996	Rayleigh, 0.988
2005 Liberal Democrat	Lognormal, 0.997	Weibull, 0.997	Folded normal, 0.989
2006 Conservative a	Lognormal, 0.999	Weibull, 0.997	Rayleigh, 0.99
2006 Conservative b	Lognormal, 0.999	Weibull, 0.996	Rayleigh, 0.99
2006 Labour	Lognormal, 0.998	Weibull, 0.997	Rayleigh, 0.989
2006 Liberal Democrat	Lognormal, 0.997	Weibull, 0.995	Folded normal, 0.987
2007	Weibull, 0.995	Lognormal, 0.994	Folded normal, 0.989
2007 Conservative	Lognormal, 0.999	Weibull, 0.997	Rayleigh, 0.994
2007 Labour	Lognormal, 0.998	Weibull, 0.996	Rayleigh, 0.989
2007 Liberal Democrat	Lognormal, 0.998	Weibull, 0.997	Rayleigh, 0.991
2008 Conservative	Lognormal, 0.999	Weibull, 0.996	Rayleigh, 0.99
2008 Labour	Lognormal, 0.998	Weibull, 0.997	Rayleigh, 0.992
2008 Liberal Democratl	Lognormal, 0.998	Weibull, 0.998	Rayleigh, 0.994
2009 Conservative	Lognormal, 0.999	Weibull, 0.996	Rayleigh, 0.993
2009 Labour	Lognormal, 0.999	Weibull, 0.995	Rayleigh, 0.992
2009 Liberal Democrat	Lognormal, 0.998	Weibull, 0.997	Rayleigh, 0.991
2010 Conservative	Lognormal, 0.999	Weibull, 0.996	Rayleigh, 0.994
2010 Labour	Lognormal, 0.998	Weibull, 0.995	Rayleigh, 0.987
2010 Liberal Democratl	Lognormal, 0.999	Weibull, 0.996	Rayleigh, 0.993
2011 Conservative	Lognormal, 0.999	Weibull, 0.996	Rayleigh, 0.993
2011 Labour	Lognormal, 0.999	Weibull, 0.996	Rayleigh, 0.994
2011 Liberal Democrat	Lognormal, 0.999	Weibull, 0.997	Rayleigh, 0.994
2012 Conservative	Lognormal, 0.999	Weibull, 0.996	Rayleigh, 0.995
2012 Liberal Democrat	Lognormal, 0.999	Weibull, 0.996	Rayleigh, 0.992
2013 Conservative	Lognormal, 0.999	Weibull, 0.997	Rayleigh, 0.996
2013 Labour	Lognormal, 0.999	Weibull, 0.997	Rayleigh, 0.996
2013 Liberal Democrat	Lognormal, 0.998	Weibull, 0.997	Rayleigh, 0.992
2014 Conservative	Lognormal, 0.999	Weibull, 0.997	Rayleigh, 0.995
2014 Labour	Lognormal, 0.999	Weibull, 0.997	Rayleigh, 0.993
2014 Liberal Democrat	Lognormal, 0.999	Weibull, 0.996	Rayleigh, 0.991
2015 Conservative	Lognormal, 0.998	Weibull, 0.998	Rayleigh, 0.996
2015 Labour	Lognormal, 0.997	Weibull, 0.997	Rayleigh, 0.991
2015 Liberal Democrat	Lognormal, 0.998	Weibull, 0.997	Rayleigh, 0.99
2016 Conservative	Lognormal, 0.998	Weibull, 0.997	Rayleigh, 0.992
2016 Labour	Weibull, 0.997	Lognormal, 0.997	Folded normal, 0.991
2016 Liberal Democrat	Lognormal, 0.998	Weibull, 0.998	Rayleigh, 0.992
2017	Lognormal 0.997	Weibull 0.996	Rayleigh 0.991
2017 Conservative	Lognormal, 0.998	Weibull, 0.997	Rayleigh, 0.992
2017 Labour	Weibull, 0.997	Lognormal, 0.997	Rayleigh, 0.991
2017 Liberal Democrat	Weibull, 0.997	Lognormal, 0.996	Folded normal, 0.991
2018 Conservative	Lognormal, 0.998	Weibull, 0.997	Rayleigh, 0.993
2018 Labour	Lognormal, 0.997	Weibull, 0.996	Folded normal, 0.989

“a” and “b” denote different speeches by the same party during the same year.

## Appendix B

The parameters for the lognormal distributions used for the description of the empirical word length distributions.

**Table A3:** Data for the USA.

Year	R^2^	*µ*	*σ*
1789	0.995	1.27	0.73
1796	0.998	1.19	0.60
1797	0.992	1.26	0.73
1798	0.989	1.29	0.73
1801	0.997	1.25	0.68
1803	0.996	1.25	0.68
1805	0.994	1.27	0.70
1805	0.996	1.28	0.68
1809	0.993	1.25	0.75
1812	0.992	1.32	0.70
1813	0.995	1.26	0.70
1815	0.993	1.23	0.73
1817	0.994	1.26	0.70
1821	0.994	1.26	0.69
1821	0.996	1.27	0.68
1825	0.993	1.27	0.71
1825	0.992	1.29	0.72
1827	0.994	1.24	0.69
1833	0.993	1.26	0.71
1837	0.991	1.29	0.72
1838	0.992	1.29	0.71
1839	0.993	1.24	0.74
1841	0.993	1.25	0.71
1842	0.993	1.23	0.73
1845	0.993	1.27	0.71
1848	0.993	1.30	0.68
1850	0.993	1.25	0.71
1851	0.993	1.23	0.71
1853	0.994	1.28	0.71
1854	0.988	1.30	0.73
1856	0.990	1.24	0.74
1857	0.995	1.28	0.68
1861	0.997	1.25	0.68
1861	0.996	1.22	0.70
1864	0.994	1.29	0.68
1865	0.992	1.30	0.68
1865	0.998	1.22	0.61
1868	0.992	1.32	0.69
1869	0.994	1.23	0.70
1873	0.995	1.21	0.71
1875	0.996	1.24	0.69
1876	0.994	1.27	0.71
1877	0.991	1.29	0.71
1877	0.992	1.27	0.72
1879	0.989	1.30	0.71
1881	0.995	1.27	0.68
1885	0.992	1.28	0.71
1889	0.995	1.28	0.68
1893	0.992	1.29	0.71
1893	0.991	1.31	0.71
1895	0.990	1.29	0.72
1897	0.992	1.27	0.70
1901	0.993	1.29	0.70
1905	0.998	1.21	0.66
1909	0.992	1.25	0.72
1913	0.998	1.20	0.65
1917	0.998	1.18	0.64
1921	0.993	1.29	0.71
1925	0.995	1.25	0.69
1929	0.993	1.29	0.72
1933	0.996	1.24	0.69
1937	0.996	1.26	0.68
1941	0.997	1.20	0.67
1945	0.999	1.16	0.66
1949	0.995	1.30	0.67
1953	0.997	1.23	0.64
1957	0.998	1.19	0.63
1961	0.998	1.22	0.62
1965	0.997	1.18	0.62
1969	0.998	1.16	0.64
1973	0.999	1.16	0.67
1977	0.998	1.20	0.65
1981	0.997	1.19	0.67
1985	0.998	1.22	0.64
1989	0.999	1.15	0.61
1993	0.997	1.23	0.64
1997	0.998	1.23	0.63
2001	0.995	1.22	0.66
2009	0.998	1.22	0.62
2013	0.998	1.25	0.63
2017	0.997	1.27	0.61
2021	0.998	1.17	0.62

**Table A4:** Data for UK.

Year	R^2^	*µ*	*σ*
1808	0.996	1.24	0.68
1814	0.996	1.21	0.70
1815	0.998	1.16	0.68
1817	0.995	1.22	0.68
1819	0.993	1.26	0.71
1827	0.996	1.10	0.71
1830	0.998	1.20	0.65
1837	0.996	1.25	0.67
1842	0.994	1.22	0.71
1853	0.995	1.21	0.68
1868	0.998	1.20	0.70
1877	0.996	1.17	0.70
1893	0.993	1.15	0.73
1895 Liberal	0.998	1.17	0.69
1896 Liberal	0.998	1.17	0.67
1897 Liberal	0.998	1.17	0.66
1897 Conservative	0.998	1.19	0.67
1899 Liberal	0.998	1.16	0.67
1900 Conservative	0.998	1.18	0.63
1901 Liberal	0.998	1.18	0.66
1902 Conservative	0.997	1.20	0.68
1903 Conservative	0.998	1.19	0.68
1903 Liberal	0.998	1.16	0.67
1904 Conservative	0.997	1.21	0.66
1905 Liberal	0.997	1.21	0.68
1906 Conservative	0.997	1.19	0.69
1907	0.998	1.14	0.69
1907 Conservative	0.998	1.21	0.67
1907 Liberal	0.998	1.18	0.67
1908 Liberal	0.998	1.17	0.66
1908 Conservative	0.998	1.20	0.66
1909 Liberal	0.998	1.17	0.67
1909 Conservative	0.998	1.18	0.66
1910 Liberal	0.998	1.17	0.68
1910 Conservative	0.998	1.18	0.66
1911 Conservative	0.998	1.17	0.65
1912 Liberal	0.997	1.17	0.68
1912 Conservative	0.999	1.15	0.64
1913 Conservative	0.999	1.16	0.63
1913 Liberal	0.997	1.18	0.69
1917	0.999	1.18	0.66
1918 Liberal	0.998	1.20	0.68
1919 Liberal	0.997	1.18	0.69
1920 Liberal	0.997	1.18	0.69
1920 Conservative	0.999	1.11	0.64
1921 Liberal	0.998	1.18	0.68
1921 Conservative	0.999	1.17	0.63
1922 Liberal	0.997	1.19	0.69
1922 Conservative	0.998	1.13	0.67
1923 Liberal	0.996	1.20	0.70
1924 Liberal	0.995	1.23	0.70
1924 Labour	0.997	1.19	0.68
1924 Conservative	0.998	1.16	0.66
1925 Liberal	0.995	1.26	0.69
1925 Conservative	0.997	1.20	0.67
1926 Conservative	0.998	1.21	0.66
1927	0.999	1.16	0.65
1927 Liberal	0.999	1.20	0.61
1927 Conservative	0.998	1.18	0.64
1928 Liberal	0.998	1.18	0.67
1928 Conservative	0.998	1.18	0.65
1929 Liberal	0.999	1.20	0.62
1929 Conservative	0.998	1.20	0.63
1930 Liberal	0.997	1.21	0.67
1932 Conservative	0.998	1.17	0.67
1932 Liberal	0.997	1.24	0.66
1933 Conservative	0.999	1.17	0.65
1934 Conservative	0.998	1.16	0.67
1935 Conservative	0.998	1.17	0.65
1936 Liberal	0.994	1.26	0.68
1937	0.997	1.19	0.67
1937 Liberal	0.996	1.22	0.68
1941 Liberal	0.998	1.23	0.65
1942 Liberal	0.996	1.24	0.67
1943 Liberal	0.997	1.24	0.66
1945 Liberal	0.996	1.23	0.67
1946 Labour	0.998	1.20	0.67
1947	0.998	1.19	0.64
1947 Labour	0.998	1.18	0.67
1948Labour	0.997	1.20	0.68
1949 Labour	0.998	1.19	0.67
1950 Labour	0.998	1.18	0.67
1951 Labour	0.998	1.17	0.67
1955 Conservative	0.998	1.18	0.65
1956 Conservative	0.998	1.19	0.66
1957	0.999	1.19	0.61
1957 Conservative	0.998	1.18	0.64
1958 Conservative	0.998	1.21	0.65
1960 Conservative	0.998	1.20	0.67
1961 Conservative	0.998	1.19	0.65
1962 Conservative	0.997	1.22	0.67
1963 Liberal	0.998	1.20	0.65
1963 Conservative	0.997	1.22	0.67
1964 Labour	0.996	1.24	0.69
1965 Labour	0.997	1.22	0.68
1965 Conservative	0.999	1.19	0.65
1966 Labour	0.994	1.28	0.69
1966 Conservative	0.998	1.19	0.64
1967	0.997	1.20	0.68
1967 Labour	0.997	1.25	0.67
1967 Conservative	0.998	1.19	0.65
1968 Labour	0.997	1.26	0.66
1968 Conservative	0.998	1.23	0.66
1969 Labour	0.997	1.24	0.65
1969 Conservative	0.998	1.18	0.64
1970 Labour	0.997	1.25	0.67
1970 Conservative	0.997	1.19	0.69
1971 Conservative	0.998	1.19	0.64
1971 Labour	0.998	1.26	0.66
1972 Conservative	0.998	1.18	0.66
1972 Labour	0.998	1.22	0.66
1973 Conservative	0.998	1.18	0.64
1973 Labour	0.997	1.26	0.65
1974 Labour	0.996	1.26	0.67
1975 Conservative	0.998	1.20	0.64
1975 Labour	0.996	1.28	0.68
1976 Conservative	0.998	1.21	0.65
1976 Labour	0.997	1.22	0.68
1977 Conservative	0.998	1.19	0.63
1997 Labour	0.998	1.25	0.65
1977 Liberal a	0.995	1.25	0.69
1977 Liberal b	0.995	1.26	0.70
1978 Conservative	0.999	1.20	0.63
1978 Labour	0.998	1.18	0.67
1978 Liberal	0.997	1.25	0.66
1979 Conservative	0.998	1.20	0.64
1979 Labour	0.998	1.19	0.66
1979 Liberal	0.997	1.24	0.67
1980 Conservative	0.998	1.22	0.65
1980 Labour	0.998	1.18	0.66
1980 Liberal	0.995	1.27	0.68
1981 Conservative	0.998	1.20	0.63
1981 Labour	0.998	1.17	0.66
1981 Liberal	0.997	1.25	0.67
1982 SDP-Liberal Alliance	0.995	1.21	0.70
1982 Conservative	0.998	1.21	0.62
1982 Labour	0.999	1.15	0.65
1982 Liberal	0.996	1.26	0.67
1983 Conservative	0.998	1.21	0.63
1983 Labour	0.998	1.16	0.65
1983 Liberal	0.996	1.25	0.67
1984 Conservative	0.998	1.21	0.64
1984 Labour	0.997	1.22	0.65
1984 Liberal	0.996	1.24	0.68
1985 Conservative	0.998	1.24	0.63
1985 Labour	0.998	1.21	0.65
1985 Liberal	0.996	1.28	0.67
1986 Conservative	0.997	1.27	0.62
1986 Labour	0.997	1.22	0.66
1986 Liberal	0.996	1.27	0.67
1987	0.996	1.30	0.65
1987 Conservative	0.997	1.26	0.63
1987 Labour	0.998	1.20	0.67
1987 SDP-Liberal Alliance a	0.997	1.24	0.66
1987 SDP-Liberal Alliance b	0.997	1.27	0.67
1988 Conservative	0.997	1.26	0.63
1988 Labour	0.996	1.25	0.67
1988 Liberal	0.996	1.23	0.68
1989 Conservative	0.998	1.23	0.62
1989 Labour	0.998	1.19	0.66
1990 Labour	0.996	1.24	0.66
1991 Conservative	0.999	1.21	0.61
1991 Labour	0.996	1.23	0.65
1992 Conservative	0.999	1.21	0.61
1992 Labour	0.997	1.22	0.66
1992 Liberal Democrat	0.999	1.20	0.63
1993 Conservative	0.999	1.20	0.60
1993 Labour	0.998	1.23	0.63
1993 Liberal Democrat	0.998	1.22	0.67
1994 Conservative	0.998	1.21	0.61
1994 Labour	0.998	1.20	0.65
1994 Liberal Democrat	0.998	1.23	0.65
1995 Conservative	0.999	1.21	0.61
1995 Labour	0.997	1.21	0.63
1996 Conservative	0.998	1.18	0.62
1996 Labour	0.999	1.16	0.64
1996 Liberal Democrat	0.998	1.22	0.62
1997	0.996	1.19	0.67
1997 Conservative	0.998	1.19	0.63
1997 Labour	0.998	1.19	0.64
1998 Conservative	0.998	1.23	0.63
1998 Labour	0.997	1.19	0.63
1998 Liberal Democrat	0.998	1.23	0.63
1999 Conservative	0.998	1.23	0.64
1999 Labour	0.998	1.20	0.63
1999 Liberal Democrat a	0.999	1.19	0.66
1999 Liberal Democrat b	0.997	1.24	0.64
2000 Conservative	0.999	1.21	0.61
2000 Labour	0.997	1.24	0.64
2000 Liberal Democrat	0.998	1.26	0.61
2001 Conservative	0.997	1.23	0.63
2001 Labour	0.997	1.24	0.64
2001 Liberal Democrat	0.997	1.26	0.65
2002 Conservative	0.999	1.21	0.60
2002 Labour	0.998	1.22	0.64
2002 Liberal Democrat	0.997	1.28	0.64
2003 Conservative	0.999	1.23	0.60
2003 Labour	0.998	1.19	0.63
2003 Liberal Democrat	0.998	1.26	0.63
2004 Conservative	0.999	1.20	0.61
2004 Labour	0.998	1.21	0.62
2004 Liberal Democrat	0.998	1.28	0.62
2005 Conservative	0.999	1.17	0.63
2005 Labour	0.998	1.21	0.64
2005 Liberal Democrat	0.997	1.24	0.65
2006 Conservative a	0.999	1.24	0.64
2006 Conservative b	0.999	1.22	0.63
2006 Labour	0.998	1.22	0.64
2006 Liberal Democrat	0.997	1.24	0.65
2007	0.994	1.32	0.70
2007 Conservative	0.999	1.17	0.60
2007 Labour	0.998	1.19	0.64
2007 Liberal Democrat	0.998	1.25	0.62
2008 Conservative	0.999	1.21	0.63
2008 Labour	0.998	1.21	0.61
2008 Liberal Democratl	0.998	1.24	0.60
2009 Conservative	0.999	1.20	0.60
2009 Labour	0.999	1.21	0.60
2009 Liberal Democrat	0.998	1.22	0.62
2010 Conservative	0.999	1.22	0.60
2010 Labour	0.998	1.18	0.64
2010 Liberal Democratl	0.999	1.22	0.61
2011 Conservative	0.999	1.22	0.60
2011 Labour	0.999	1.18	0.59
2011 Liberal Democrat	0.999	1.23	0.60
2012 Conservative	0.999	1.19	0.58
2012 Liberal Democrat	0.999	1.21	0.62
2013 Conservative	0.999	1.20	0.58
2013 Labour	0.999	1.15	0.58
2013 Liberal Democrat	0.998	1.22	0.62
2014 Conservative	0.999	1.18	0.59
2014 Labour	0.999	1.19	0.60
2014 Liberal Democrat	0.999	1.23	0.61
2015 Conservative	0.998	1.21	0.59
2015 Labour	0.997	1.24	0.62
2015 Liberal Democrat	0.998	1.19	0.63
2016 Conservative	0.998	1.23	0.62
2016 Labour	0.997	1.28	0.63
2016 Liberal Democrat	0.998	1.21	0.62
2017	0.997	1.25	0.62
2017 Conservative	0.998	1.22	0.62
2017 Labour	0.997	1.29	0.63
2017 Liberal Democrat	0.996	1.25	0.67
2018 Conservative	0.998	1.22	0.62
2018 Labour	0.997	1.25	0.65

“a” and “b” denote different speeches by the same party during the same year.
